# ATXN3: a multifunctional protein involved in the polyglutamine disease spinocerebellar ataxia type 3

**DOI:** 10.1017/erm.2024.10

**Published:** 2024-09-25

**Authors:** Esperanza Hernández-Carralero, Grégoire Quinet, Raimundo Freire

**Affiliations:** 1 Fundación Canaria Instituto de Investigación Sanitaria de Canarias (FIISC), Unidad de Investigación, Hospital Universitario de Canarias, La Laguna, Santa Cruz de Tenerife, Spain; 2 Instituto de Tecnologías Biomédicas, Centro de Investigaciones Biomédicas de Canarias, Facultad de Medicina, Campus Ciencias de la Salud, Universidad de La Laguna, Santa Cruz de Tenerife, Spain; 3 Faculty of Health Sciences, Universidad Fernando Pessoa Canarias, Las Palmas de Gran Canaria, Spain

**Keywords:** ATXN3, spinocerebellar ataxia type-3, ubiquitin hydrolase, neurodegeneration, polyglutamine neurodegenerative disorders, proteostasis, ubiquitin proteasome system, chromatin organization, DNA damage response

## Abstract

ATXN3 is a ubiquitin hydrolase (or deubiquitinase, DUB), product of the *ATXN3* gene, ubiquitously expressed in various cell types including peripheral and neuronal tissues and involved in several cellular pathways. Importantly, the expansion of the CAG trinucleotides within the *ATXN3* gene leads to an expanded polyglutamine domain in the encoded protein, which has been associated with the onset of the spinocerebellar ataxia type 3, also known as Machado–Joseph disease, the most common dominantly inherited ataxia worldwide. ATXN3 has therefore been under intensive investigation for decades. In this review, we summarize the main functions of ATXN3 in proteostasis, DNA repair and transcriptional regulation, as well as the emerging role in regulating chromatin structure. The mentioned molecular functions of ATXN3 are also reviewed in the context of the pathological expanded form of ATXN3.

## Ataxin-3 protein structure and domains

**Figure 1.**
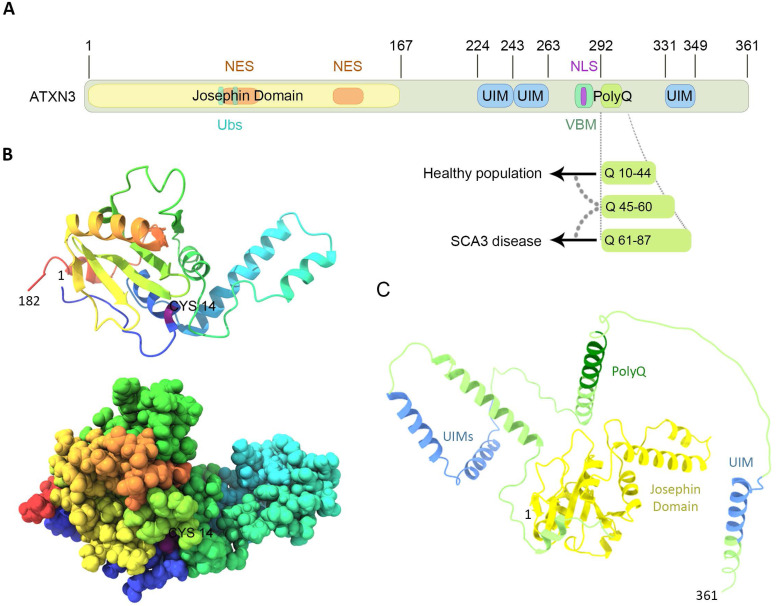
Domain organization and molecular structure of ATXN3. (A) Schematic representation of the most common isoform of ATXN3 (UniProt P54252-2). ATXN3 is composed of an N-terminal catalytic domain (Josephin domain, JD), followed by a C-terminal tail containing three UIMs and a polyglutamine sequence of variable length (PolyQ). ATXN3 holds a nuclear localization signal (NLS) located upstream of the PolyQ region and two nuclear-export signals (NES) within the JD. The VBM sequence around the PolyQ track is responsible for the binding to VCP/p97. (B) Cartoon (upper panel) and space-filling (lower panel) models of the Josephin domain crystal structure (PDB ID: 1YZB). Cysteine 14 (Cys14) is labelled in purple. (C) Cartoon predicted model of ATXN3 (AlphaFold ID: P54252-F1). Functional domains are indicated as in (A).

Ataxin-3 (ATXN3) protein is encoded by the *ATXN3* gene that contains 11 exons (Ref. 1); alternative splicing of the *ATXN3* gene can lead to different transcripts/protein isoforms (Refs 2, 3, 4). The canonical form of ATXN3 has 361 amino acids (UniProt P54252-2) (Fig. 1A), containing an N-terminal Josephin domain (JD), responsible for the ubiquitin hydrolase catalytic activity (Fig. 1A and B). ATXN3 also contains a flexible C-terminal region, harbouring three ubiquitin interacting motifs (UIMs) (Fig. 1C). UIMs are known to bind ubiquitin moieties but can also function as recruitment points for interactors (Ref. 5). The C-terminal domain of ATXN3 additionally contains a polyglutamine (PolyQ) region, encoded by the CAG codon, of variable length. The extension of the CAG repeats on the *ATXN3* gene leads to the translation of an expanded PolyQ-tract form of ATXN3 (ATXN3-PolyQ), that is associated with the onset of the most common form of spinocerebellar ataxia worldwide, spinocerebellar ataxia type 3 (SCA3), a dominant inherited neurodegenerative disease (Fig. 1A) (Refs 6, 7, 8, 9, 10). Also, in between the UIMs and the PolyQ domain, a VCP binding motif (VBM) is situated (Fig. 1A), responsible for the interaction of ATXN3 with its partner VCP/p97 (Cdc48 in yeast), hereafter called p97, an AAA + ATPase known to facilitate the degradation of polyubiquitinated substrates in the proteasome (see below). Several regions of the ATXN3 structure have been individually solved by crystallography, as for example the C-terminus of the protein (Ref. 11), the JD (Ref. 12) and the tandem UIM domains (Ref. 13).

The JD is highly conserved from human to yeast, and mutation of the catalytic cysteine residue (Cys14) abolishes the protease activity of ATXN3 (Fig. 1B) (Refs 12, 14). The JD contains two contiguous but distinct ubiquitin binding sites (Ubs, Fig. 1A), which are important for the enzymatic activity of ATXN3. Ubs1 is contiguous to the active site and is required for the cleavage of ubiquitin molecules as it allows the correct positioning of the C-terminus of ubiquitin into the active site. In contrast, only a minor role of Ubs2 in the ubiquitin hydrolase activity of ATXN3 has been suggested (Refs 15, 16). ATXN3 is able to bind both lysine 48 (K48)- and lysine 63 (K63)-linked ubiquitin chains, but with preference to cleave K63-linked ubiquitin chains of at least four ubiquitin molecules. This specificity seems to be determined by the UIMs, since the JD alone preferentially cleaves K48-linked ubiquitin chains (Refs 14, 17, 18).

ATXN3 also contains different motifs that regulate its localization. Typically, ATXN3 localizes diffusely throughout the cell. However, diverse stimuli can induce different posttranslational modifications (PTMs) that affect its re-distribution and function (Refs 19, 20, 21). For example, Casein kinase 2-dependent phosphorylation of serine 340 and 352 within the third UIM are important for ATXN3 nuclear localization and stability (Ref. 21).

The subcellular localization of ATXN3 can also change by active translocation between the nucleus and the cytosol, regulated by nuclear import and export signals, since ATXN3 contains a putative nuclear localization signal located at amino acid 282–285, immediately N-terminal to the PolyQ region; and two nuclear exporting signals within the JD, positioned at amino acids 77–99 and 141–158 (Fig. 1) (Refs 6, 20, 22, 23). Proteotoxic stress, induced by heat or oxidative stress, leads to ATXN3 accumulation in the nucleus. This translocation into the nucleus is independent of its catalytic activity and its UIMs, whereas phosphorylation of serine 111 in the JD seems to be essential (Ref. 24). However, the activity of ATXN3 plays a role in its general subcellular distribution, as catalytic inactive ATXN3 was less enriched in nucleus compared to the active version of ATXN3 (Ref. 25).

## Regulation of ATXN3 ubiquitin hydrolase activity and stability

ATXN3 is a ubiquitin hydrolase (or deubiquitinase, DUB), an enzyme that cleaves the ubiquitin molecules from protein substrates (Ref. 18). Interestingly, ATXN3 was the first reported DUB in which ubiquitination directly regulates its catalytic activity. Indeed, monoubiquitination of ATXN3 within its JD, which is enhanced by proteotoxic stress, increases its ubiquitin hydrolase activity by locking its catalytic domain into an active mode without changing its overall structure nor its specificity towards ubiquitin chains (Refs 26, 27). This monoubiquitination at lysine 117 of ATXN3 is sufficient for the ubiquitination-dependent activation of the DUB (Ref. 28). ATXN3 can also undergo polyubiquitination by several ubiquitin E3 ligases with preference for K48-linked ubiquitin chains, and proteasome inhibition has been shown to stabilize ATXN3 (Refs 29, 30). In addition, several studies on cellular models supported a regulation of ATXN3 through autophagy, although for wild-type ATXN3 (ATXN3-WT) proteasome clearance remains more important (Refs 29, 31). For example, the E3 ligase PJA1 promotes ATXN3 degradation by the proteasome and also by autophagy (Ref. 31). The Ubs2 moreover controls ATXN3 turnover as protein levels are reduced when this site is mutated. Indeed, Ubs2 mediates the interaction of ATXN3 with the proteasome-associated proteins hHR23A and hHR23B, thereby preventing proteasomal degradation of ATXN3 (Refs 16, 17, 32, 33). E3 ubiquitin ligase Gp78 additionally promotes the polyubiquitination and degradation of ATXN3, targeting it for endoplasmic reticulum-associated protein degradation (ERAD), a cellular pathway that degrades misfolded proteins (Ref. 34).

Surprisingly, although the catalytic inactive version of ATXN3 undergoes more extensive ubiquitination than ATXN3-WT, it is degraded at slower rates by the proteasome than the WT version (Ref. 25). ATXN3 interacts with the proteasome shuttle protein p97, and this interaction was shown to be reduced in case of a catalytic inactive version of ATXN3, which can explain the lower rate of its proteasomal degradation (Ref. 25). Interestingly, although its UIMs mediate ATXN3 ubiquitination, the inactivation of these motifs does not alter ATXN3 degradation, suggesting that UIM-mediated polyubiquitination of ATXN3 modulates its function instead of stability (Ref. 35).

## ATXN3 and proteostasis

ATXN3 plays important roles in protein homeostasis or proteostasis. Protein degradation is essential for proteostasis and cell survival, as it replenishes the amino acid pool for *de novo* protein synthesis, allowing cells to adapt to their intra- and extracellular environment. In normal conditions, misfolded proteins are either refolded properly by molecular chaperones or degraded by the ubiquitin-proteasome system (UPS). When these systems do not function well, misfolded proteins accumulate, often forming oligomers and small aggregates that are potentially cytotoxic. ATXN3 is involved in different proteostasis pathways, including UPS, the ERAD, autophagy and fibrillar aggregates formation.

The UPS is the principal mechanism for the turnover of short-lived and damaged proteins in eukaryotic cells (Ref. 36). As ubiquitination controls the fate of proteins, the edition and disassembly of polyubiquitin chains, as well as recycling ubiquitin, is critical for cellular homeostasis. As ubiquitin hydrolase, ATXN3 is essential for the tight regulation of the UPS (Refs 37, 38, 39). Supporting the role of ATXN3 in the UPS, the expression of a catalytically inactive version of ATXN3 in cells results in accumulation of ubiquitinated proteins, a phenotype also observed in cells from ATXN3-knock out mice (Refs 35, 40, 41). In contrast, the overexpression of ATXN3 results in a significant reduction of protein ubiquitination in different cell lines (Ref. 18). A large panel of specific substrates of ATXN3 has been characterized and some are described hereafter.

ERAD is a quality control system in the secretory pathway responsible for degrading misfolded proteins, within the unfolded protein response (UPR), and unassembled polypeptides of protein complexes. Proteins targeted for ERAD are deglycosylated, ubiquitinated, extracted from the endoplasmic reticulum and degraded by the proteasome. A central player essential for extracting substrates from the endoplasmic reticulum to the cytosol is p97, a homo-hexameric barrel-like protein that forms multiple complexes and sub-complexes with associated cofactors that confer p97 specificity to multiple substrates and cellular pathways (Refs 15, 42, 43). These p97 complexes recognize target proteins and extract these ubiquitinated substrates using the p97 ATPase activity. Finally, ubiquitinated proteins are either presented to the proteasome for degradation or they become substrates of DUBs to recycle ubiquitin (Refs 44, 45). ATXN3 is involved in the cellular protein quality control system by interacting with p97, acting as a cofactor. In unperturbed conditions, ATXN3 collaborates with p97 in the process of ERAD, assisting p97 in the extraction and unfolding of proteins for subsequent delivery to the proteasome. Additionally, p97 was shown to be an activator of the DUB activity of ATXN3 (Refs 46, 47, 48).

**Figure 2.**
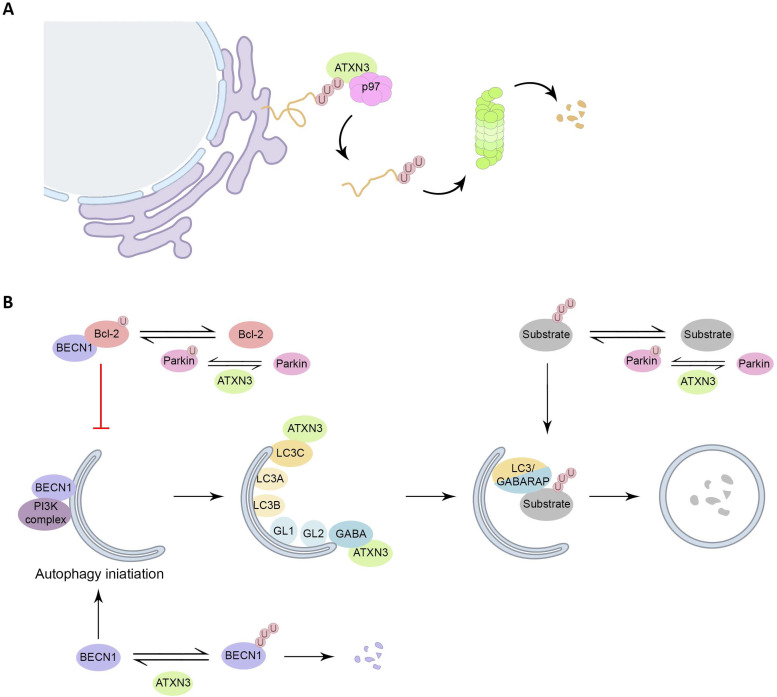
Roles of ATXN3 in proteostasis. (A) ATXN3 is required for efficient ERAD, assisting p97 in the extraction and unfolding of proteins for subsequent delivery to the proteasome. (B) Interaction of Bcl-2 with BECN1 inhibits autophagy initiation, Parkin monoubiquitinates Bcl-2, leading to an increase on Bcl-2 levels. ATXN3 counteracts Parkin E3 activity, by interacting with it, and hampering the auto-ubiquitination of Parkin. Also, ATXN3 removes ubiquitin chains from BECN1, leading to its stabilization and stimulation of autophagy. ATXN3 interacts with LC3C and GABARAP in early stages of autophagy, promoting autophagy. ATXN3 also regulates the activity of the E3 ligase Parkin, important for the ubiquitination of autophagy substrates and the autophagy inhibitor Bcl-2.

The interaction of ATXN3 with p97 occurs between the VBM of ATXN3 (Fig. 1A) and the N-terminal domain of p97, in a nucleotide-dependent way, as ATXN3 and p97 associate in the absence of nucleotide or the presence of ATP, but not in the presence of ADP (Ref. 49). The binding of ATXN3 to p97 results in a decreased interaction between p97 and other cofactors. In turn, this sequestration of p97 by ATXN3 reduces the retrotranslocation of substrates from the endoplasmic reticulum, resulting in a decreased degradation of ERAD substrates in cellular models. Therefore, ATXN3 has been suggested to regulate the flow through the ERAD pathway by adjusting the rate of extraction of ERAD substrates (Refs 50, 51) (Fig. 2A).

Autophagy is the main degradation process that clears long half-life proteins, protein complexes and organelles. Autophagy is an important clearance route for aggregate-prone proteins like PolyQ-expanded proteins. This system can efficiently sequester misfolded proteins to facilitate their clearance once the UPR and UPS have failed. In general, short-lived and soluble misfolded proteins are targeted by UPS, whereas long-lived and insoluble protein aggregates are eliminated by autophagy (Refs 15, 52). Several links between ATXN3 and autophagy have been described (Fig. 2B). First, ATXN3 interacts specifically with the autophagy key proteins LC3C and GABARAP, members of the LC3/GABARAP proteins family in mammalian cells. This transient regulatory interaction takes place via two interacting regions localized within the JD during the earlier stages of the autophagic pathway, supporting a role of ATXN3 during the autophagy process (Ref. 53). Additionally, in vitro assays have shown that ATXN3 can interact with the E3 ligase Parkin through ATXN3 UIM domains, which hampers the auto-ubiquitination of Parkin, known to be essential for its E3 activity (Ref. 54). Parkin has two important functions in the autophagy system. On the one hand, Parkin monoubiquitinates the autophagy inhibitor Bcl-2, resulting in increased levels of Bcl-2 (Ref. 55). On the other hand, Parkin-mediated K63-polyubiquitination has been described as a signal for targeting misfolded proteins to autophagy (Ref. 56).

Another ATXN3 substrate is BECN1, a critical component of the class III phosphatidylinositol 3-kinase complex that generates phosphatidylinositol-3-phosphate, a signalling lipid responsible for the recruitment of downstream autophagy factors, needed for the early formation of the autophagosome (Refs 57, 58). ATXN3 was furthermore proposed to act as an adaptor to link polyubiquitinated substrates to the dynein motor protein, thus facilitating the retrograde transportation of the substrates to the aggresomes, cellular perinuclear structures formed by the aggregation of misfolded proteins due to overwhelming protein degradation in the cell. Supporting this hypothesis, depletion of ATXN3 leads to decreased aggresome formation (Refs 53, 59).

Finally, ATXN3 has been shown to assemble into amyloid fibrils, which are insoluble fibres resistant to degradation. In this context, an aggregation-prone region located in the JD of ATXN3 leads to formation of sodium dodecyl sulphate (SDS)-soluble protofibrils in cells expressing ATXN3 (Ref. 60).

## ATXN3 and the DNA damage response

Recently, ATXN3 has been suggested to play a significant role in DNA repair mechanisms to maintain genomic stability. The integrity of the human genome is constantly threatened by a variety of endogenous and environmental factors and cells need to repair the damage suffered in their genetic material to faithfully transmit the information it contains to the next generation. To do so, eukaryotes have evolved an extremely efficient and complex response to DNA lesions, collectively known as the DNA damage response (DDR).

DNA strand breaks (SBs), both single-strand (SSBs) and double-strand breaks (DSBs), are among the most toxic and mutagenic lesions in mammalian genomes. These lesions, if not accurately repaired, can lead to a wide range of mutations or genome aberrations that threaten cell or organism viability (Ref. 61). Mammalian cells execute two main DSB repair pathways: non-homologous end joining (NHEJ) and homologous recombination (HR). NHEJ directly ligates the DSBs ends, and it is generally considered to be an error-prone DSB repair pathway. On the other hand, HR restores the lost DNA sequence at DSB sites, using an undamaged DNA sequence of the identical sister chromatid as a template. Because of the use of the sister chromatid, HR is only functional in S and G2 phases, while NHEJ is active throughout the cell cycle (Refs 62, 63).

**Figure 3.**
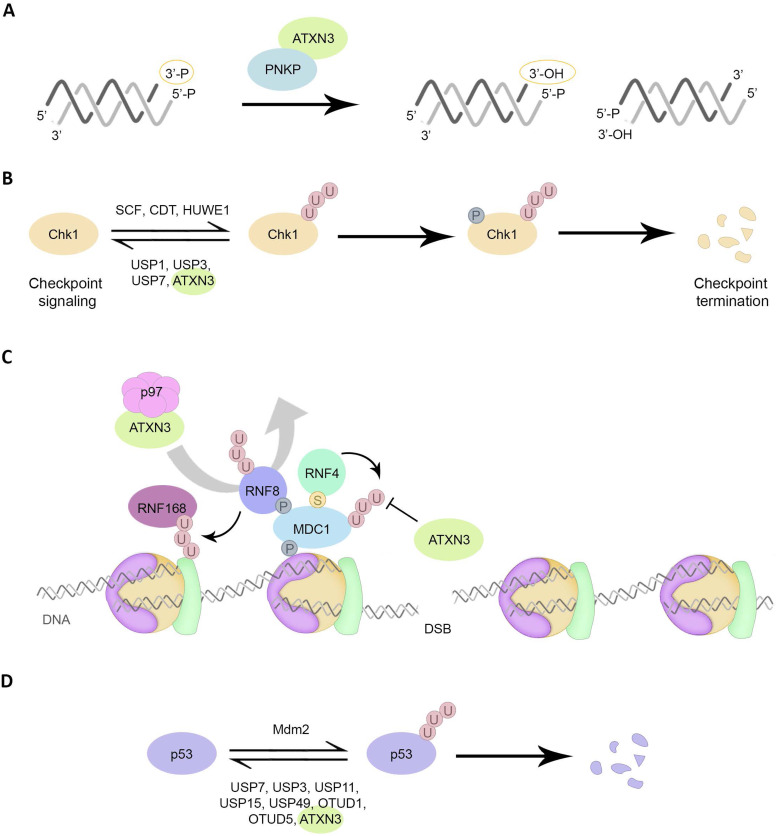
Involvement of ATXN3 in genome integrity maintenance. (A) The association between ATXN3 and PNKP promotes the 3′ phosphatase activity of PNKP, removing the 3′-P group, and consequent SB repair. (B) Under unperturbed conditions and upon DNA damage, ATXN3 interacts with Chk1 and antagonizes polyubiquitination and degradation of Chk1, facilitating DNA damage checkpoints and repair. Under prolonged replicative stress, phosphorylation of Chk1 results in dissociation of Chk1 from ATXN3. (C) During the early phase of DSB response, ATXN3 antagonizes RNF4-induced polyubiquitination and subsequent chromatin-release of MDC1. By prolonging the residence time of MDC1 at chromatin, ATXN3 ensures that the DDR is accurately activated. The p97 ATXN3 complex promotes RNF8 extraction from damage sites to avoid RNF8 overaccumulation and inhibition of NHEJ, ensuring proper DSBs repair. (D) p53 ubiquitination is mainly governed by the E3 ligase Mdm2 and leads to proteasomal degradation of p53. Among other DUBs, ATXN3 stabilizes p53 by direct deubiquitination of the protein.

In order to repair DNA SBs, the conventional 3′-OH (hydroxyl) and 5′-P (phosphate) ends must be restored for gap-filling and DNA ligation to occur. The DNA end-processing enzyme polynucleotide kinase 3′-phosphatase (PNKP) removes the 3′-P group and catalyses the phosphorylation of 5′-OH termini, and is therefore involved in the repair of both SSBs and DSBs (Refs 64, 65). ATXN3 helps DNA repair by interacting with PNKP and stimulating DNA 3′-end-processing activity, thereby preventing the accumulation of DNA SBs (Fig. 3A). Indeed, ATXN3-deficient cells shown decreased PNKP activity accompanied by an increase in DNA SBs (Refs 66, 67). Moreover, as observed in both cellular models and *Drosophila* mutants, in the absence of ATXN3, PNKP inactivation results in the accumulation of persistent 3′-P termini, while RNA polymerase II, which is normally deubiquitinated by ATXN3, becomes poly-ubiquitinated and subsequently degraded. This causes transcriptional blockage and unrepaired DSB breaks in the transcribed genome (Ref. 68).

Interestingly, ATXN3 regulates Chk1, a central kinase in the DDR. Chk1 is activated by different types of DNA lesions and replication stress, delaying cell cycle progression to facilitate DNA repair. During S phase, activated Chk1 phosphorylates Cdc25A phosphatase, inducing its degradation, thereby inhibiting origin firing and replication fork progression. Additionally, Chk1 suppresses excessive origin firing during the unperturbed cell cycle by negatively regulating the Treslin/TICRR complex and Cdc45, both critical initiators of DNA replication (Refs 69, 70). Chk1 activity is modulated through several PTMs, including ubiquitination, as Chk1 levels are tightly controlled by proteasomal degradation. Specifically, two Cullin RING ubiquitin ligase E3 complexes have been described to control Chk1 levels: SCF (Skp1-Cullin1-Fbx6) and CDT (CDT2-Cullin4A-DDB1) (Refs 71, 72, 73, 74, 75, 76). HUWE1 was additionally identified as an E3 ubiquitin ligase that directly regulates Chk1 stability (Ref. 77). ATXN3 is one of the described ubiquitin hydrolases for Chk1, thereby essential for the maintenance of steady-state levels of Chk1, prolonging its half-life and checkpoint signalling. Moreover, under prolonged replicative stress, phosphorylation of Chk1 at Ser345 results in dissociation from ATXN3, allowing Chk1 proteasomal degradation and checkpoint termination (Ref. 78) (Fig. 3B).

ATXN3 also regulates initial stages of the DDR after DSBs induction. The ubiquitin-like modifier SUMO is a critical PTM engaged in the regulation of the DSB response (Ref. 79). DNA damage-induced SUMOylation results in the recruitment of the SUMO-targeted ubiquitin ligase RNF4 to DNA lesions. There RNF4 facilitates ubiquitin-dependent removal of chromatin-associated MDC1, a mediator in this response, and loading of replication protein A and Rad51 onto ssDNA, promoting DSB repair (Refs 80, 81, 82). In this context, ATXN3 is recruited to DSBs independently of its UIMs, but in a SUMOylation-dependent manner, where it counteracts RNF4 through its DUB activity by regulating MDC1 ubiquitination (Fig. 3C). This way, ATXN3 prevents the premature removal of MDC1 from DNA lesions and ensures efficient recruitment of DSB-response factors downstream MDC1, such as RNF8, RNF168, BRCA1 and 53BP1. The opposing and balanced activities of RNF4 and ATXN3 thereby consolidate robust MDC1-dependent signalling and repair of DSBs by NHEJ and HR (Ref. 83). Poly [ADP-ribose] polymerase 1-mediated poly(ADP-ribosyl)ation (PARylation) has also been shown to play a role in this process, as SUMO-dependent recruitment of ATXN3, but not RNF4, to DSBs requires DNA damage-induced PARylation. This could explain how ATXN3 and RNF4, despite their opposing activities, have simultaneous roles in DDR mechanism (Ref. 84).

In addition to their collaboration in the process of ERAD, ATXN3 and p97 cooperate in the control of DDR-protein levels (Ref. 85). The RNF168/ubiquitin/53BP1 cascade is essential to mediate NHEJ and is functionally distinct from the RNF8/ubiquitin/BRAC1/Rad51-governed HR repair pathway. Consequently, for correct DSB repair, the levels of RNF8 and RNF168 must be tightly regulated. RNF168 protein levels are indeed strictly regulated by two E3 ubiquitin ligases, UBR5 and TRIP12, and a DUB, USP7 (Refs 86, 87). On the other hand, the homeostasis of RNF8 is regulated by p97 and ATXN3 (Ref. 88). Specifically, under genotoxic stress, when RNF8 is rapidly recruited to sites of DNA lesions, the p97-ATXN3 machinery stimulates the extraction of RNF8 from chromatin (Fig. 3C) to balance DNA repair pathway choice and promote cell survival after DSB formation.

Last, a link between ATXN3 and the tumour suppressor p53 was found. p53 is a crucial protein in genomic stability maintenance and often mutated in cancer cells, highlighting the importance of a tight regulation of p53 for supporting normal cellular functions (Refs 89, 90, 91). p53 is mainly regulated at the protein level, and its stability is usually kept low due to ubiquitination by several E3 ubiquitin ligases that target p53 for proteasomal degradation, with MDM2 as the major E3 ubiquitin ligase involved. Among other DUBs (Refs 92, 93, 94, 95, 96, 97, 98), ATXN3 has been described to bind native and polyubiquitinated p53 to repress its UPS-dependent degradation (Fig. 3D). Thus, ATXN3 deletion destabilizes p53, resulting in lack of p53 activity and functions (Ref. 99).

## ATXN3 and chromatin organization

Using different cellular models, our group recently demonstrated regulation of global chromatin organization by ATXN3, leading to effects on different DNA-related processes such as replication and transcription. This work describes the ATXN3-mediated recruitment of the histone deacetylase 3 (HDAC3) to the chromatin, which promotes a more compact chromatin state. In the absence of ATXN3, HDAC3 is not properly localized in to the chromatin, changing chromatin accessibility, which leads to defects in different processes where DNA is used as a template, such as DNA replication and transcription (Ref. 100). Specifically, DNA replication timing was altered due to the activation of more replication origins, as they were more exposed to the binding of the replication machinery. Moreover, transcription was globally increased, and nuclear and nucleolar morphologies were disrupted because of the more accessible state of the chromatin in the absence of ATXN3. Interestingly, this novel function of ATXN3 upon chromatin organization occurs independently of its catalytic activity, supporting the role for this DUB as a scaffold protein (Ref. 100).

ATXN3 has previously been described to function in transcriptional control but the studies focused on certain gene promoters (Refs 101, 102). For instance, ATXN3 has been shown to interact with HDAC3 and participate in its recruitment to promoter regions, repressing the transcription of the *MMP-2* gene in a UIM-dependent manner (Ref. 101). Our data show a more global effect on chromatin organization, which could also explain previously described roles of ATXN3 in transcription (Ref. 100). Indeed, ATXN3 additionally has been shown to interact with several transcriptional components, such as the TATA-binding protein-associated factor TAFII130, the cAMP response element (CRE) binding protein (CREB)-binding protein (CBP), p300 and p300/CBP-associated factor (PCAF), the nuclear corepressor receptor NCoR and HDAC6 (Refs 101, 102, 103, 104, 105, 106, 107, 108). CBP and p300 are transcriptional coactivators with histone acetyltransferase (HAT) activity that have important roles in transcription and have been described to be regulated by some PolyQ-containing proteins (Refs 109, 110, 111). CBP and p300 mediate transcriptional activation by many signal-dependent transcription factors, including CREB. In response to cAMP, CREB bound to the CRE enhancer is phosphorylated, resulting in the recruitment of CBP/p300 and eventually the transcriptional activation of cAMP-responsive genes. The C-terminal domain of ATXN3 binds CBP, p300 and PCAF coactivators and represses their transcriptional activity (Ref. 106).

Apart from exogenous DNA damaging agents, genome integrity can be threatened by spontaneous damage, such as that caused by reactive oxygen species (ROS) derived from normal metabolism. In this context, the forkhead box class O (FOXO) family transcription factors are involved in various cellular processes, including resistance to cellular oxidative stress. ATXN3 has been described as a redox-sensitive transcriptional coactivator of the superoxide dismutase 2 (*SOD2*) gene within the FOXO-mediated oxidative stress response, as an increased binding of ATXN3 and FOXO4 to the SOD2 promoter is associated with upregulation of the antioxidant enzyme SOD2 in response to oxidative stress (Ref. 112).

## Molecular basis of SCA3, behaviour of pathogenic ATXN3

As already introduced, SCA3, also known as Machado–Joseph disease (MJD), is a hereditary neurodegenerative disorder with autosomal dominant transmission. The SCA3 and ataxias in general are motor coordination dysfunctions that affect gait, balance, speech and vision. Multiple neuronal systems are affected in SCA3 patients, including the cerebellum, the brainstem and some cranial nerves, the basal ganglia and the spinal cord. SCA3 is associated with an abnormal and unstable expansion of a CAG tract that encodes for glutamine in the open reading frame of the *ATXN3* gene. The CAG repeats number ranges from 10 to 44 in the healthy population and from 61 to 87 in SCA3 patients, whereas CAG repeats between 45 and 60 are associated with an incomplete penetrance of the disease (Fig. 1). As with other PolyQ diseases, a higher number of CAG repeats is associated with an earlier onset of the disease. Although the ATXN3-PolyQ is expressed ubiquitously in various tissues and cell types, mutation of this protein seems only to induce neuronal dysfunction. Moreover, while clinical heterogeneity has been observed among SCA3 patients, ataxia is the main clinical hallmark (Refs 10, 113, 114).

Even though SCA3 is caused by abnormal PolyQ expansion in ATXN3, the underlying mechanism causing the disease remains elusive. One of the most relevant neuropathological features of SCA3 is the presence of intraneuronal inclusions of mutant ATXN3 proteins in postmortem patient brains, due to the change in conformation of ATXN3. Interestingly, these inclusions were not only detected within brain regions described to be affected by neurodegeneration but also in regions that typically are not injured in the disease (Refs 115, 116, 117). These observations lead to a model where the observed toxicity is attributed to soluble oligomers that precede inclusion body formation in the aggregation process. Moreover, the protein aggregation observed due to the expression of ATXN3-PolyQ is thought to sequester essential proteins involved in protein quality control and transcription, causing cytotoxicity and cell death (Refs 9, 113, 118, 119, 120).

## ATXN3-PolyQ and proteostasis

As mentioned, ATXN3-WT plays important roles in cellular proteostasis. In this section, we discuss the differences with the pathological version of ATXN3 (ATXN3-PolyQ) in these processes. First, at the molecular level, the ubiquitination status of ATXN3-PolyQ is not significantly different than the normal ATXN3, except at lysine 8 where the PolyQ-expanded variant shows increased ubiquitination, although this higher lysine 8 modification in the pathological variant has no impact on its stability (Ref. 29). It remains to be explored whether this modification of lysine 8 in ATXN3-PolyQ affects the development of SCA3. ATXN3 itself is regulated by autophagy and the proteasome and ATXN3-PolyQ is as efficiently degraded as ATXN3-WT (Ref. 35). However, immunohistochemistry analysis has shown that most of the ATXN3-containing inclusion bodies from SCA3 patients were immunopositive for subunits of 19S regulatory particles of the proteasome, whereas only a subset of them were also positive for subunits of the 20S catalytic core of the proteasome (Ref. 121). This dissociation of the primary subcomplexes (20S and 19S particles) of the proteasome suggests that a perturbation in the proteasomal machinery that degrades misfolded and damaged proteins, in addition to important regulatory molecules, could contribute to the pathology in SCA3 patients. In fact, expression of ATXN3-PolyQ compromises the functionality of the UPS resulting in increased levels of proteasome substrates (Ref. 14). And, although in vitro studies suggested that the DUB activity of ATXN3-PolyQ is not affected (Refs 18, 122), the insolubility and immobile nature of the inclusions may make ATXN3-PolyQ less available to properly recognize all substrates for deubiquitination, behaving in practical terms as a catalytic inactive mutant (Ref. 113). As a result, misfolded and damaged proteins, as well as important regulatory molecules that escape the UPS degradation, can accumulate and trigger the neurotoxicity observed in SCA3.

As E3 ubiquitin ligases have been implicated in the pathogenesis of PolyQ diseases, different studies have attempted to identify enzymes with this activity that could alleviate the cellular toxicity of ATXN3-PolyQ. Interestingly, overexpression of the Cullin-1-based ubiquitin E3 ligase FBXO33 increases the solubility of ATXN3-PolyQ and reduces protein toxicity in a cellular model (Ref. 123). Also, transient overexpression of the E3 ubiquitin ligase ITCH, which interacts with ATXN3-PolyQ, alleviates cytotoxicity induced by aggregation of misfolded proteins, supporting the importance of ubiquitination in SCA3 (Ref. 124).

ATXN3-PolyQ also has an impact on the ERAD response. Although the elongation or truncation of the PolyQ region has no impact on the interaction with p97, ATXN3-PolyQ sequesters endogenous p97 into insoluble aggregates, causing a decrease of endoplasmic reticulum retrotranslocation and degradation of ERAD substrates (Ref. 125). The accumulation of misfolded proteins in the endoplasmic reticulum may therefore contribute to SCA3 disease pathogenesis (Ref. 51).

Defects in the ubiquitination system have been related to several neurodegenerative diseases including SCAs, Parkinson's disease (PD) and Huntington's disease (Ref. 126). Interestingly, SCA3 can present clinical and neuropathological features of PD and the affected brain regions can overlap (Ref. 127). As mentioned before, Parkin, mutations which are a common cause of PD, acts as an E3 ubiquitin ligase (Ref. 128). Although both ATXN3-WT and ATXN3-PolyQ can deubiquitinate Parkin, the pathological form of ATXN3 promotes the clearance of Parkin via the autophagy pathway, implicating increased Parkin turnover in the pathogenesis of SCA3, possibly explaining some of the Parkinson-like features observed in the disease (Ref. 129).

As previously mentioned, ATXN3-WT has been shown to form amyloid fibrils. Pathological ATXN3-PolyQ is able to perform an extra irreversible step of assembly, making the amyloid-like fibrils large and SDS-resistant (Ref. 130). Although some early studies suggested that aggregation into inclusion bodies could be a protective mechanism by which the cell deals with PolyQ proteins (Refs 131, 132, 133), the propensity of ATXN3-PolyQ to form aggregates is considered as a characteristic hallmark of SCA3, forming toxic intraneuronal inclusions (Refs 115, 134, 135). Other reports suggest that the proteolytic cleavage of PolyQ proteins generate shorter fragments containing the expanded PolyQ region (Ref. 135). As aggregations of these smaller PolyQ oligomers have been found to be enriched in brains of SCA3 mouse models and patients, they have been proposed to be responsible for the neuronal cytotoxicity (Refs 135, 136, 137). Interestingly, expression of exogenous ATXN3-WT suppressed the ATXN3-PolyQ-associated neurodegeneration in flies (Refs 138, 139). ATXN3-WT additionally reduced aggregate formation by the ATXN3-PolyQ variant expressed in human cells (Ref. 3), supporting a protective role of ATXN3-WT in proteostasis in a SCA3 disease context.

## Pathological ATXN3 and DNA repair

Defects in the DDR are reported to be associated with various genetic diseases accompanied by neurodegeneration, such as ataxia telangiectasia, xeroderma pigmentosum, trichothiodystrophy and Cockayne syndrome (Ref. 120). We described before how ATXN3 contributes to the DDR. Although no hypersensitivity to DNA-damaging agents has been reported in SCA3 patients, there is an impact of the ATXN3-PolyQ version in DNA repair, observed as a dramatic increase in focus formation of phosphorylated H2AX and 53BP1 in cells expressing ATXN3-PolyQ, SCA3 mouse brains and brain sections from SCA3 patients, indicating an accumulation of DNA damage (Refs 67, 140).

**Figure 4.**
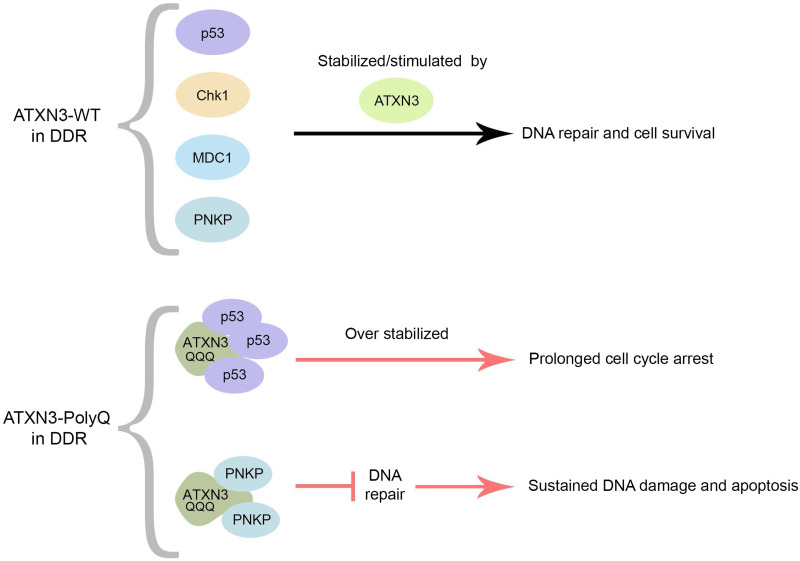
Normal and pathogenic roles of ATXN3 in the DDR. ATXN3-WT regulates the kinetics of early and late events in the DDR process. As a DUB, ATXN3 stabilizes MDC1, Chk1 and p53 through the removal of polyubiquitin chains, and promotes PNKP phosphatase activity, stimulating DNA repair and cell survival. On the other hand, ATXN3-PolyQ inhibits PNKP-dependent DNA repair and prolongs p53-signaling through enhanced p53-binding and stabilization. This leads to increased activation of pro-apoptotic pathways and cell vulnerability to DNA damage.

At the cellular level, the pathological form of ATXN3 affects several aspects of the DDR. While ATXN3-WT stimulates PNKP activity, PNKP has been found to be recruited into the insoluble ATXN3-PolyQ aggregates with a marked reduction of its kinase catalytic activity in SCA3 brain (Fig. 4). The interaction of PNKP with ATXN3-PolyQ, and its sequestration in aggregates, therefore leads to an abrogation of DNA repair efficacy, to the subsequent chronic activation of the ATM and the downstream pro-apoptotic p53-dependent signalling pathways in SCA3 (Refs 66, 67).

Although ATXN3-WT regulates the stabilization and pro-apoptotic function of p53, the expansion of its PolyQ track impedes the dissociation between ATXN3 and p53. ATXN3-PolyQ consequently dysregulates and enhances the stability of p53 compared to ATXN3-WT (Fig. 4), which, in turn, causes more p53-dependent apoptosis/necrosis in mammal cells, zebrafish and mouse SCA3 models (Ref. 99).

## Impact of ATXN3-PolyQ on chromatin structure and transcription

ATXN3-PolyQ has been reported to alter transcriptional activity by impairing repressor activity of the *MMP-2* gene promoter, when compared with ATXN3-WT (Ref. 101). Moreover, ATXN3-PolyQ has a reduced capacity to activate FOXO-mediated SOD2 expression during oxidative stress, abrogating the expression of SOD2 and disrupting the clearance of ROS (Ref. 112). Interestingly, given the high level of oxygen consumption in the nervous system, ROS-induced cytotoxicity and oxidative DNA damage are believed to contribute to SCA3 pathogenesis. The fact that ATXN3-WT binds to components of the transcriptional machinery, as well as the transcriptional changes reported in animal models of SCA3 in the pre-symptomatic phase, support the hypothesis of a general transcriptional dysregulation as an initiating factor of SCA3, although the specific molecular basis underlying this dysregulation remained elusive (Refs 103, 141, 142, 143). Recently our group published data that help to understand this general transcriptional effect due to the role of ATXN3 in chromatin organization. While in non-pathogenic conditions ATXN3-WT promotes the compaction of the chromatin, thereby constraining RNA synthesis, the expression of ATXN3-PolyQ promotes chromatin accessibility, which leads to a general increase in transcription, a similar phenotype as the one observed in the absence of ATXN3 (Ref. 100). Consistently, expression of ATXN3-PolyQ is unable to reverse the open status of the chromatin in the absence of ATXN3, which is compatible with increased global transcription, together with changes in the expression of specific genes, for example previously mentioned *MMP-2* (Refs 100, 101, 103, 105, 143, 144).

Our group found that ATXN3-PolyQ sequesters HDAC3, impeding correct HDAC3 chromatin localization and thereby promoting a more open state of the chromatin (Ref. 100). Consistently, ATXN3-PolyQ expression in ATXN3-knock out cells is not able to rescue nucleolar dysfunctions. Our data are compatible with previous reports describing the impaired binding of the extended PolyQ tract of ATXN3 to chromatin in specific genes (Ref. 101), although ATXN3-PolyQ is still able to interact with HDAC3 (Ref. 100). This could mean that in SCA3, ATXN3-PolyQ may lead to the global expression of normally repressed genes (Ref. 100) instead of regulating a subset of genes, as described before (Refs 101, 105).

In the last years, the levels and enzymatic activity of HATs and HDACs, the enzymes responsible for regulating acetylation homeostasis within the nucleus, have emerged as essential for neuronal viability, with an important role in neurodegenerative conditions. It has for example been shown that treating neurons with HDAC inhibitors or overexpressing HATs leads to cell death (Refs 145, 146, 147, 148, 149), highlighting the importance of the acetylation balance for neuronal viability in non-pathological conditions. Therefore, the mislocalization of HDAC3 upon ATXN3-PolyQ expression points to another molecular mechanism that could participate in the onset or the progression of SCA3.

## Summary

ATXN3 has been extensively studied for decades as the protein causing SCA3, the most common inherited ataxia worldwide. The expansion of a CAG repeat in the coding region of the *ATXN3* gene leads to the translation of an expanded PolyQ tract in the C-terminus of the protein that has been associated to neurotoxicity. Unfortunately, the exact molecular role of ATXN3-PolyQ in the pathogenesis of SCA3 remains elusive and the disease therefore cureless. Here we have highlighted the molecular functions of ATXN3-WT and discussed the similarities and differences with the pathological variant.

ATXN3 is a deubiquitinating enzyme, of which the ubiquitin hydrolase activity is activated by its monoubiquitination at lysine 117. ATXN3 is further regulated by ubiquitination on other residues and degraded by the proteasome and autophagy. The ubiquitin status of ATXN3 seems not affected by the expansion of the PolyQ tract, neither is its intrinsic catalytic activity or its degradation rate by proteolytic pathways.

ATXN3 is involved in different pathways controlling proteostasis. As a ubiquitin hydrolase, it cleaves ubiquitin from proteins before degradation, for reuse. Although both ATXN3-WT and ATXN3-PolyQ can deubiquitinate the E3 ligase Parkin, associated with PD, ATXN3-PolyQ increases the turnover of Parkin, which could possibly contribute to some of the Parkinson-like features in SCA3. ATXN3 additionally plays a role in ERAD, acting as a p97 cofactor. Furthermore, ATXN3 is involved in the DDR, for example by the stabilization of p53 and recent work from our group demonstrated a role for ATXN3 in the regulation of the chromatin status by recruiting HDAC3 to the chromatin.

Importantly, both ATXN3-WT and ATXN3-PolyQ are aggregate-prone proteins and can form short protofibrils due to self-association of the JD. However, an irreversible second step of aggregation occurs only with ATXN3-PolyQ, producing long fibrils resistant to SDS. This irreversible aggregation is toxic for the cell and causes functional alterations that can affect its interacting partners. For example, ATXN3-PolyQ enhances p53 stabilization as compared to ATXN3-WT and thereby triggers a prolonged cell cycle arrest and neuronal dysfunction. In addition, whereas ATXN3-WT controls the efficient recruitment of HDAC3 to the chromatin and in this manner participates in heterochromatin formation, ATXN3-PolyQ sequesters HDAC3, causing mislocalization of HDAC3 and a more open state of the chromatin, impacting on processes such as replication and transcription.

In conclusion, ATXN3 has essential roles in various cellular pathways. Dysregulation of some of these pathways upon the expansion of the ATXN3 PolyQ tract, followed by the self-assembly of the protein, may explain the origin of the SCA3 pathogenesis, as supported by the correlation between the severity of the symptoms and the PolyQ tract length.
